# Unconventional combinations of prospective parents: ethical challenges faced by IVF providers

**DOI:** 10.1186/s12910-017-0177-x

**Published:** 2017-02-28

**Authors:** Robert Klitzman

**Affiliations:** Columbia University, 1051 Riverside Drive #15, New York, NY 10032 USA

**Keywords:** Fertilization, in vitro, Reproduction, Infertility, Ethics, Policy, Decision making, Gamete donation, Families, Discrimination

## Abstract

**Background:**

Professional guidelines have addressed ethical dilemmas posed by a few types of nontraditional procreative arrangements (e.g., gamete donations between family members), but many questions arise regarding how providers view and make decisions about these and other such arrangements.

**Methods:**

Thirty-seven ART providers and 10 patients were interviewed in-depth for approximately 1 h each. Interviews were systematically analyzed.

**Results:**

Providers faced a range of challenges and ethical dilemmas concerning both the content and the process of decisions about requests for unconventional interfamilial and other reproductive combinations. Providers vary in how they respond — what they decide, who exactly decides (e.g., an ethics committee or not), and how — often undergoing complex decision-making processes. These combinations can involve creating or raising the child, and can shift over time — from initial ART treatment through to the child’s birth. Patients’ requests can vary from fully established to mere possibilities. Arrangements may also be unstable, fluid, or unexpected, posing challenges. Difficulties emerge concerning not only familial but social, combinations (e.g., between friends). These arrangements can involve blurry and confusing roles, questions about the welfare of the unborn child, and unanticipated and unfamiliar questions about how to weigh competing moral and scientific concerns — e.g., the autonomy of the individuals involved, and the potential risks and benefits. Clinicians may feel that these requests do not “smell right”; and at first respond with feelings of “yuck,” and only later, carefully and explicitly consider the ethical principles involved. Proposed arrangements may, for instance, initially be felt to involve consanguineous individuals, but not in fact do so. Obtaining and verifying full and appropriate informed consent can be difficult, given implicit familial and/or cultural expectations and senses of duty. Social attitudes are changing, yet patients’ views of these issues may also vary, based on their cultural backgrounds.

**Conclusions:**

These data, the first to examine how clinicians make decisions about unconventional reproductive arrangements, highlight several critical ethical questions and ambiguities, and variations in clinicians’ responses. While several professional guidelines exist, the current data highlight additional challenges, and have vital implications for improving future guidelines, practice, education and research.

**Trial registration:**

Not applicable.

## Background

Assisted Reproductive Technologies (ART) are enabling increasing numbers of adults to consider and pursue unconventional reproductive combinations and roles that often pose ethical challenges for clinicians. Three types of parental combinations, other than “traditional” arrangements of married heterosexual couples, have been examined and termed “nontraditional” — gay and lesbian parents and single parents by choice [1, 2], and are now commonly accepted in many regions in the US and other Western countries. However, requests for several additional types of reproductive arrangements have been described, including gamete donations between various combinations of family members, such as to and from male and female siblings, and mothers and daughters serving as surrogates for each other [3] — that I here term “unconventional.”

These various unconventional types of combinations have received far less attention, and may present challenges that both resemble and differ from each other. Sister-to-sister egg donation, for instance, can provide an important option to many women [4]. For an infertile female patient who, to have a child, requires a third party (e.g., an egg donor or gestational surrogate), using a sister, rather than anonymous third party can allow the patient to still have a genetic connection with the child, and dramatically reduce costs. Yet these arrangements can also pose challenges [4]. Certain other familial arrangements can, however, raise various additional problems related to real or perceived biological consanguinity, or confused roles for eventual offspring or others [5, 6]. Incest taboos and bans on reproduction between closely-related individuals are widespread among societies, partly due to concerns about possible birth defects. As more individuals seek ART and as costs of gamete (especially egg) donation and gestational surrogacy remain relatively high, such patients will no doubt increasingly continue to seek cheaper, unconventional approaches.

In vitro fertilization (IVF) clinics vary considerably in whether they accept such unusual combinations, and if so, which. In 1998, 90% and 80% of US ART clinics would accept eggs from family members and friends, respectively; and 60% would accept family members’ sperm [7]. These ART clinic directors were also generally more restrictive than their clinics’ policies regarding many of these issues — e.g., 14.8% would personally restrict use of eggs from family members, which was twice as restrictive as their clinics’ policies [7]. Clinic directors may be more conservative than their clinics’ policies due to fear of litigation and “dynamics of inter-clinic politics” [7], raising questions about how clinic policies are created, and how often these are followed. In a survey of US ART program directors, using hypothetical questions, 67% would allow a male patient’s brother without children to donate sperm, 29% would allow a male patient’s father to donate sperm, and 18% would allow a female patient’s mother to donate eggs [8]. Among US obstetrician-gynecologists in 2008–2009, regarding nontraditional arrangements, more generally, 17% would discourage single parents, and 14% would discourage unmarried or lesbian patients. Male and religious respondents were more than two and three times, respectively, more likely to discourage ART use by these groups [9].

The American Society for Reproductive Medicine (ASRM) has stated, “The use of intrafamilial gamete donors and surrogates is generally ethically acceptable when all participants are fully informed and counseled” [3], but recommends prohibition of two related individuals each providing gametes. A brother donating sperm to fertilize his sister’s egg, for instance, would be incestuous [3, 10]. Moreover, “Child-to-parent arrangements are generally unacceptable, and parent-to-child arrangements are acceptable in limited situations” [3]. Other arrangements may appear to outsiders to be incestuous when that is not in fact the case (e.g., a mother carrying a fetus for her daughter or a brother donating sperm to his sister who is, along with her husband, infertile). The European Society of Human Reproduction and Embryology (ESHRE) has published similar guidelines [11].

Some critics have argued that with intrafamilial donations and surrogacy, truly voluntary decisions are impossible, due to emotional and financial pressures in families — family members may feel coerced or unduly influenced to participate [3]. Moreover, once the child is born, familial roles and relationships can prove complicated, especially if the child encounters medical, psychological, or developmental problems, for which parents may then blame these arrangements [3]. Dilemmas also arise regarding what to tell the offspring about these relationships. Hence, ASRM’s Ethics Committee guidelines concerning use of family members as gamete donors or surrogates emphasize needs to avoid coercion or undue influence of family members involved, and needs for caution, especially with relationships that may trigger questions in others’ minds about possible incest [3]. ASRM also recommends that clinics develop policies and procedures; that some combinations may be acceptable while others are not because of consanguinity or lack of adequate informed consent; and that providers prohibit child-to-parent donation, carefully counsel and screen all participants, may deny certain requests, and should have family members obtain appropriate legal consultation [3]. ASRM also has issued recommendations concerning age maximums for women using donor eggs [12, 13]. But this organization does not monitor or enforce its guidelines, and most clinics have been not following key guidelines from the organization — e.g., concerning selection of donors and compensation [14].

Critical questions thus arise of how clinicians in fact view and make these decisions. A few prior studies have explored aspects of how providers make decisions about ethically-challenging areas. Ehrich et al., for instance, conducted group discussions with staff at one UK clinic, affiliated with a hospital and partly providing National Health Service (NHS) treatment, concerning two types of embryo selection — based on sex and carrier status — and suggested that these two types of embryo selection challenged notions of individual autonomy, and that staff balance patient autonomy against other values. These researchers argued that a model of “relational autonomy” [15], that emphasizes the socially-embedded, non-individualism of moral agents [15–17] is more suitable than the concept of individual autonomy — though aspects of this approach have been debated [17]. However, these researchers did not examine other types of issues that arise in ART clinics, such as unconventional combinations of potential parents. 

Gerrits et al. [18] examined several types of ethically-sensitive requests addressed by a formal, regularly scheduled bi-monthly multi-disciplinary ethics committee in a Dutch hospital-based infertility clinic at an institution that has Roman Catholic roots, and is ‘rooted’ in a Catholic community (e.g., where commercial surrogacy and anonymous sperm donation are strictly forbidden). These researchers examined 29 requests, including 8 patients who were carriers of genetic disease, 5 with serious mental or psychiatric problems, 3 elder men, 3 with cancer, 2 in unstable relationships, and 8 others, including 1 case of a couple where the partners might have a close consanguineous relationship (though no further details about this last case were provided). The committee drew on personal views and ethical principles, upheld the reproductive autonomy of most couples, and helped diminish the likelihood that decisions were shaped by a physician’s individual feelings — seeking consultation, scientific evidence, more medical information, test results, referrals to other clinics, and informed consent. Yet the array of cases examined — from genetic disease to psychiatric problems to unstable heterosexual marriages — vary widely and may thus in fact often be addressed differently.

Frith et al. interviewed UK clinicians, mostly from 5 clinics affiliated with the NHS and with hospitals, and found that these providers often had difficulty naming any “ethical issues” when asked [19]. These authors dichotomized ethical concerns that arose into one of two categories, involving either “controversial”, or “settled morality” of “mundane” everyday practice; and theorized that clinicians drew a boundary between these two categories, to avoid a “take-over” by “outside” influences, challenges and control [19]. Yet crucial questions arise of what specific ethical principles and/or issues are involved in such ethical cases, and how these then get addressed or resolved — whether other categories or types of ethical issues surface besides these two (e.g.,﻿ perhaps﻿ related to the specific content of cases), and if so, what.

Moreover, while these three prior studies on ethical issues in ART examined, respectively, on one clinic in the UK, one clinic in the Netherlands, and mostly five clinics in the UK, questions emerge of whether these processes and decisions may vary across different clinics and countries, and if so, how. Indeed, in the US, 73.5% of clinics are not university or hospital affiliated, but rather are “free standing” [20], and thus may not have formal ethics committees. Moreover, new types of requests will no doubt emerge as the field and social norms continue to evolve.

Many critical questions thus remain regarding how providers in fact view and make decisions about various types of proposed unconventional parenting arrangements — e.g., what specific kinds of challenges and difficulties they confront, and how they respond to these. Strikingly, no studies have been published of how providers in fact perceive and make decisions about these particular challenges and dilemmas.

Thus, as part of an in-depth qualitative interview study of ART providers and patients, exploring attitudes and experiences concerning several critical aspects of IVF (e.g., sex selection, upper age limits of prospective mothers, numbers of embryos implanted, reductions of multi-fetal pregnancies, and insurance coverage [21–25]), issues concerning unconventional combinations of prospective parents repeatedly arose, and were thus examined, given the absence of prior research on this topic. This paper presents these data, focusing on how providers make decisions about these issues.

## Methods

As depicted in Table 1, and presented elsewhere [21–25], 37 in-depth telephone interviews of approximately 1 h each were conducted and subsequently analyzed with 27 ART providers and 10 patients. Interviews were systematically analyzed.

**Table 1:** Characteristics of sample

	Male	Female	Total
PHYSICIANS	14	3	17
Physicians who also are patients	0	1	1
Type of Practice			
University-affiliated	5	1	6
Private Practice	9	2	11
OTHER ART PROVIDERS (e.g., nurses, mental health providers)	1	9	10
Other providers who also are patients	0	3	3
PATIENTS	1	9	10
TOTAL	16	21	37

Because no earlier research has been published probing how IVF providers and patients make decisions about unconventional combinations of parents, qualitative methods were used, since these can best assay the full range and types of views, interactions and behaviors involved, and can inform later quantitative research. Qualitative methods have been employed successfully to elucidate key aspects of patient’s views and behaviors regarding other aspects of ART, including patients’ disclosures of donor eggs [26].

Geertz [27] has encouraged examining aspects of people’s lives, contexts, and desires not by imposing theoretical frameworks, but by seeking to grasp individuals’ own perspectives, through their own voices to gather a “thick description.” In the present study, methods adapted key aspects of “Grounded Theory” [28] — e.g., “constant comparison” of data from different contexts to gauge differences and similarities, and examine whether these suggest hypotheses. This approach yields new analytic categories, and assesses them for reasonableness. The PI (Principal Investigator) repeatedly considered how interviewees differed and resembled from each other, and the cultural, social and medical milieus and factors involved. Grounded Theory entails both deductively drawing on frameworks from previous research and theories, and inductively building from the data to larger patterns and themes.

### Participants

Interviewees included 27 ART providers — 17 physicians (MDs) (including 1 physician-patient [MD-PT], and 10 other providers [OPs] (e.g., mental health providers and nurses, including 3 other provider-patients [OP-PTs]) — and 10 patients [PTs]. Recruitment occurred through ASRM meetings, word-of-mouth, and listservs. Clinicians were recruited, too, through national ASRM meetings (e.g., mental health professional and PGD interest groups). The PI approached these attendees to see if they may be interested in enrolling in a survey. If yes, the PI then sent them details. The majority of those approached agreed to participate. The PI also used a mental health listserv that include﻿s about 60 clinicians (though some are inactive); 15 replied, and the first 8 were then enrolled. Interviews were conducted until “saturation” was achieved (i.e., “the point at which no new information or themes are observed in the data” [29]). Respondents came from throughout the US. Clinicians frequently discussed interactions with many patients and colleagues. Patients often described interactions with clinicians and other patients. Interviewees received a detailed information sheet about the study, on the basis of which they gave informed consent verbally over the phone, which the PI then recorded. Interviews were conducted by phone since these individuals were located across the US. The IRB approved this consent procedure.

### Instruments

The semi-structured survey/questionnaire (see Table 2 ﻿for sample questions), drew on previous research, and probed patients’ and clinicians’ perspectives and decisions regarding unconventional combinations of parents, and other critical aspects of IVF.

**Table 2:** Sample questions for providers

• What challenges do you face in your work as an ART provider? o How do you address these challenges? • Have you faced challenges concerning non-traditional combinations of patients? If so, when and how? What has been difficult about these situations? What did you do? How did you make these decisions? • How do you view these issues? • How have your patients approached these issues? • Do you have any additional thoughts about these issues?	

### Data analysis

Interviews were audio-recorded, and transcriptions and initial analysis of transcripts were done during the period when the interviews were held to optimize and inform later interviews. After all interviews were finished, later analyses were performed in two parts, primarily by trained research assistants (RAs) and the PI. First, they independently read subsets of transcripts to probe factors that affected interviewees’ perspectives, determining recurrent issues which were then given assigned codes. The PI and RAs read the interviews, coding portions of text to determine “core” codes or categories (such as requests for treatment by unconventional combinations of prospective parents, and instances of providers making decisions about these cases). Names of codes were written next to each portion of the transcript to describe the issues presented. The PI and RAs then collaborated to integrate these separately-developed codings into a single framework. A coding manual was then organized, defining the codes and exploring any differences until consensus was achieved. New codes that did not fit into the original framework were explored, and the codes were adjusted when appropriate.

The PI and RAs then independently analyzed the data to identify the main sub-codes (or subcategories) and spectrums of variation in each of the main codes. They integrated these sub-categories that each coder identified into a single set of “secondary” categories and a refining set of core categories. These sub-codes included, e.g., providers’ and patients’ instances of decisions based on “yuck” responses, and decisions made formally by committee.

Codes and sub-codes were then used in analysis of all of the interviews. To ensure coding reliability, two coders analyzed each interview. More than one code was used when needed. Differences and similarities between participants were examined, exploring variations within categories, and factors involved. Disagreements were probed through closer examination until consensus was achieved. Earlier and later codings were regularly compared for accuracy and consistency. Illustrative quotes appear below to provide readers with a sense of the range of responses and the richness of the data.

## Results

**Fig. 1:**
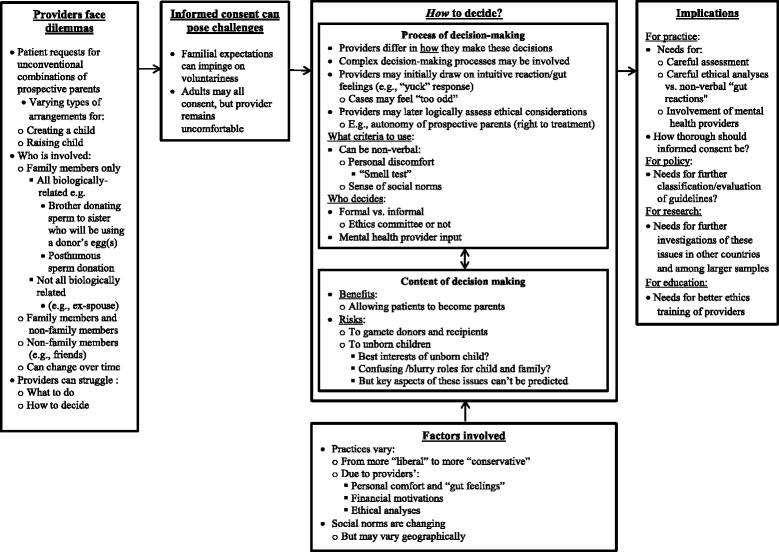
Issues concerning how clinicians make decisions about unconventional reproductive arrangements

In brief, as outlined in Fig. 1, and described more fully below, providers faced a range of challenges and dilemmas concerning both the content and the process of decisions about requests for unconventional interfamilial and other reproductive combinations. Providers varied in how they responded to such requests — what they decided, who exactly decides, and how. These combinations can create and/or raise the child, and can shift over time — from initial ART treatment through the child’s birth. Requests for these arrangements can also vary from fully established to mere possibilities (e.g., including patients’ contingency plans — e.g., if one member of the combination dies before the child reaches 18). In describing these ethically challenging cases, inter-related themes emerged, concerning the types of cases and the ways these providers made these decisions, illustrating several issues simultaneously. Thus, as seen below, the categories presented below are not rigid, but rather closely allied.

### Types of combinations

#### Unconventional social combinations

Providers grapple with not only intrafamilial donations, but a variety of other complex “informal or non-conforming social relationships” [MD#13] that may be interfamilial and inter-generational, or socially unusual in other ways. As this male physician said, “Unusual, nonconforming social relationships are one of the most challenging issues.” [MD#13]

Providers commonly encounter proposed arrangements that they had not previously anticipated or considered. Clinicians may struggle with unusual combinations involving adults with complex social as well as biological connections. Unusual social procreative arrangements were logistically possible, but could create psychological and ethical conflicts in the future.

A couple is not divorced, but he’s living and sexually intimate with another woman, not his wife. He wants to have a kid with this girlfriend. His wife, however, will carry the baby. His current girlfriend couldn’t or didn’t want to carry the baby because she’s a model. The ex-wife will do it because he’s paying her a ton of money. But you couldn’t make that up! [MD#13]

Clinicians may thus encounter unanticipated scenarios about which they have to make decisions. These planned arrangements can be hard because of concerns that the roles and responsibilities of the adults involved are unclear or can cause confusion.

In unconventional combinations, providers usually ask for careful legal contracts (“We require them to see lawyers, and go to counseling, and we require documentation” [MD#13]); but making these decisions can still be hard.

Patients’ reproductive arrangements can also change in unexpected ways over time. Clinicians might start treating a patient who has a relatively conventional arrangement that then becomes unconventional, posing dilemmas. Members of a couple may become unsure whether they want to stay together, and/or change their reproductive wishes and proposed arrangements.

We look at the environment the baby is going to be brought into — whether that’s going to be stable. Some women come in; and one cycle they’re sexually intimate with somebody, and the next cycle, they’re not sure they still want to be a parent with them. One month, they do, and the next month, they’ll want them just as the sperm donor. [MD#13]

#### Concerns about raising, as well as creating the child

Clinicians face challenges concerning unusual intrafamilial combinations of individuals involved in not only creating, but raising the eventual child. As one female nurse said,

A 65-year-old professor who is retiring wants us to help him pick an egg donor, and is going to use a surrogate. But who’s going to raise this baby? He’s got it all planned out. He’s going to retire to where his sister lives, and she’s going to raise it for hm. She is 63, and knows nothing about this. We say: “No.” [OP#6]

### Challenges with informed consent

In these situations, informed consent can pose several challenges. Family members may agree, not because they are explicitly coerced, but because of complex long-standing psychological relationships, involving perceived moral obligations, familial obligations or guilt. Inter-generational donation can pose particular but not unique challenges. As one female mental health provider described,

A mother gets remarried, wants another baby, and wants her daughter to be a donor. And the daughter says “yes,” because she feels she can’t say “no” to mom. Doctors shouldn’t do upward generational donation. It’s an informed consent and relationship issue. Plenty of mothers will do things for their daughters, but that’s a more natural, motherly act. Japanese grandmothers carry babies for their daughters since that country does not approve of egg donation except in those kinds of situations. [OP#5]

As this interviewee suggests regarding Japanese grandmothers, practices and cultural norms regarding these issues may also vary across cultures, related to differing laws and moral perspectives.

Still, the full voluntariness and thus autonomy of all the individuals involved can be hard to gauge completely, since donors may sense familial expectations or feel somewhat conflicted, though they may ultimately still be willing to proceed.

A sister is going to donate for her sister. You meet the donor sister and she tells you that she doesn’t want to do it, but “just can’t say ‘No’” to her sister. Or she tells you that she wants to do it, but she is acting like she doesn’t. [OP#5]

Clinicians can thus face difficulty and uncertainty in responding when they sense that one of the participants may have reservations. Thus, mental health providers and others can serve important roles, screening donors, and assessing motivations, to ensure that participation is wholly voluntary; yet challenges can still surface because donors may say yes, but nevertheless appear reluctant or unsure.

In other situations, family members may all fully agree to a plan; yet providers may nonetheless refuse. Informed consent can be necessary, but not sufficient, since providers may be concerned, too, about the best interests of the future child, who obviously can’t consent. As one male physician said,

One couple I turned down: the husband was 75 and the wife was 60 and they wanted to use a gestational surrogate and select for a male because he did not have a male offspring. He said, “My daughter will take care of the baby if something should happen to us.” I said, “This is not if, but when.” He was a wonderful guy. At the age of 75, some neuron fired in his head and said, “You don’t have a male offspring.” So, he wanted to get a donor egg and a gestational surrogate, so he could sire this male child. His daughter agreed she would take care of the baby. So in effect I’m producing a child for the 40-year-old-daughter, that’s going to be sired by his sperm. It was too odd for me, so we backed out. [MD#2]

This case illustrates several issues. The family members may all consent to arrangements that nonetheless pose several ethical problems. Moreover, providers may refuse to treat patients due to concerns about not only creating, but raising the child. Clinicians may also have difficulty articulating the explicit ethical issues involved, which may rather simply seem “too odd,” highlighting how providers may have certain implicit intuitive moral thresholds, on the basis of which they make decisions, and may refuse. But they may not be wholly able to define or articulate these criteria or thresholds in advance, and instead only or most clearly recognize these limits when encountering them. In addition, cases may feel unacceptable not because of one single aspect, but several together — in this instance, the age of parents, the sex selection and the child being raised, at least partly, by another family member. This case may appear extreme in certain regards, but providers often described encountering such unusual requests, to which they needed to respond.

### How to decide

Providers differ in how they make these decisions — who exactly decides, what criterion they use and how. In confronting these requests, clinicians often face challenges in determining not only whether to offer treatment, but how to decide — how to weigh the various considerations involved — frequently undergoing a process from initial disgust to more careful assessment, and at times agreement. Clinicians may initially oppose a particular arrangement because they feel intuitive moral revulsion; but they may later carefully and logically reconsider, explicitly articulating and weighing the competing ethical principles entailed. Providers may feel instinctual moral disgust that they may only subsequently question. As a female nurse said, “In some cases, the ‘yuck factor’ will get into play before they think about it, so they just say, ‘No.’” [OP#6]. For instance, a man may want to donate sperm to his twin sister and husband who need both an egg and sperm donor. This arrangement may initially appear to constitute incest, but after close analysis, does not:

The patient is in her 40s and single, and needs donor eggs to conceive. Can she use her twin brother as a sperm donor? Everybody initially went, “Ewwww.” Then we went to the ethics committee and hashed it out. We all said, “Well, why not?” It was her gene pool. It wasn’t incest.” She was using donor eggs, and her brother and his wife were very accommodating. She gave birth to a baby fathered by her brother, and it was lovely. [OP#6]

Providers may thus undergo a process over time in coming to accept certain types of arrangements. Moreover, this provider, working in a medical center-affiliated clinic, consulted an ethics committee, though providers in private free-standing clinics tended not to do so.

Many clinicians may try to address these issues logically and systematically, going through very clear and rigorous evaluative steps, but may nonetheless still draw at least partly on instinct, and use “the smell test.” As a male physician explained,

First, we ask if this is medically reasonable. Then, is it in the best interest of each patient? Thirdly, is it in the best interest of the child? Fourthly, does it pass a smell test in our profession? Fifthly, how does it relate to practice and ethical guidelines? Sixth, does it make sense for us as a practice to do this, or should we send them somewhere else? Then, what kinds of medical, psychological, financial, legal management, are we going to need? And ethics problems. So, we go through all those aspects, and try to determine what to do. Eight out of 10 times, we tell people, “If you do these things, then we’ll be happy to provide service. But if you don’t want to do these things, we can’t provide you this service.” [MD#4]

In other cases, initial moral intuitions and the “yuck” factor may nonetheless prevail. For instance, regarding the case mentioned above of the request of a 75-year-old man and his 60-year-old wife for a male child whom the couple’s daughter would eventually raise, the physician was sympathetic, but torn. This male doctor continued,

I would describe myself, politically, as closest to being libertarian — who are we to judge? If it’s not dangerous, than whose life are you endangering? That was a tough case because if everybody really agreed to it, I was not endangering anyone’s life. This baby would have an unusual start, but would presumably have a stable home and everything else would be okay. [MD#2]

Nonetheless, in the end, this doctor refused because the request still didn’t feel instinctively “right”:

It was a non-verbal, personal kind of decision. I said, “No. It’s our policy not to work with old couples,” but that was a cop-out — although ultimately that was the problem. Producing babies for a couple that was that old, with no realistic expectation that either one of them would be around, was not right! We discussed it, and all decided we were uncomfortable. [MD#2]

Clinician may thus decide, not based on explicit analyses per se, but on their personal “comfort.” Providers may also consult each other on such thorny cases. Providers may refuse to offer treatment because of an initial non-verbal response, and then search for a reason that may not in fact fit the specific case precisely, but seem to serve as a legitimate excuse.

### Factors Involved

A range of factors, including personal and political attitudes and beliefs, professional characteristics, institutional structures and financial motives can shape whether and how much providers will support such requests. Multiple reasons can be involved — pro and con. Providers could simply respect and follow the patient’s autonomy and wishes, but may also consider the best interests of the future progeny. The proposed arrangement could be difficult for the child, blurring familial roles in ways that, this physician felt, lay too far outside perceived norms. About the case above, involving elderly prospective parents, the physician continued,

It would still be traumatic for the child, who would be raised by the grandparents for as long as they could do it, and then the child would go back to the de facto mother — the daughter. [MD#2]

This physician then drew on his staff’s feelings and perceptions of social norms.

That didn’t make any sense to me. So I’m producing a child for a mom sired by her father. I thought it stepped outside the norm. [MD#2]

Clinics may vary in their decisions, partly due to different views of norms concerning parenting arrangements, which are changing, though not necessarily equally throughout the country. Instead, attitudes can vary by geographic region, fueling inter-clinic differences. As a male physician said, “Another practice does all kinds of alternative family building pathways; but we’re a pretty buttoned-up practice.” [MD#9]

Providers’ own financial motives may also play roles, balanced against degrees of possible ethical concern. As a female nurse said, “In some cases, it’s all about the money. They’ll do pretty much anything as long as somebody pays for it. Those are the practices that make the big, newsworthy mistakes.” [OP#6]

### Who decides

Clinics may vary due partly to who makes these determinations — what their personal and professional roles are. As mentioned above, some clinics consult formal, external ethics committees, while others do not. Physicians may discuss such ethically difficult cases with their staff, formally or informally. Most clinics also draw on mental health providers (“We get opinions from psychologists” [MD#13]). These assessments can frequently help in identifying and addressing problematic arrangements. As a female nurse described, “A couple of times a year, a psych session leads us to not going forward.” [OP#6] Yet clinics may vary in how, and to what extent they use such mental health providers. Who decides may affect what is decided. This nurse also observed, “It depends on the clinic, and who’s running it, and what their personal views are.” [OP#6] As she suggests, clinicians may also differ in whether and why they will treat certain cases, and how they and others perceive possible motives.

## Discussion

These data, the first to examine several critical aspects of how providers experience, view and make decisions about unconventional reproductive arrangements, suggest that clinicians face several dilemmas concerning both the process and content of these decisions — whether to treat such cases, who should decide, and how. These requests fall across a wide spectrum from acceptable to problematic, varying along several parameters, based on the patients’ respective functional roles (from donating gametes to carrying the fetus to raising the child), relationships (from biologically-related family members, to non-biologically related family members, to friends) and perceived norms. These combinations differ in certain regards, but also pose similar ethical challenges, involving blurry and confusing roles, questions about the ongoing welfare of the unborn child, and unanticipated and unfamiliar questions about how to weigh the competing moral, personal, social and scientific concerns involved.

While ASRM guidelines address a few aspects of these issues, these data highlight several additional critical sets of challenges and ambiguities, and make several contributions to the existing literature. While Gerrits et al. [18], Ehrich et al. [15] and Frith et al. [19] focused on aspects of ethical decision-making in one Dutch, one UK, and essentially five UK clinics, respectively, the present data examined a wider range of clinics, and suggest considerable variations among both clinics and countries, in how they approach these issues. Infertility clinics in the US and many other countries are far less regulated than in the UK and the Netherlands. The Human Fertilisation and Embryology Authority (HFEA) regulations, for instance, may constrain clinics far more than do guidelines and regulations in many other countries [30], including the US, where ASRM guidelines are often looser and less unenforced [14].

These data also reveal differences in *who* makes these decisions. Many of these clinics lack formal ethics committees, and providers wrestle with these issues, making decisions on their own, or occasionally consulting with other physicians or staff, engaging in processes that range from formal to informal, and vary based partly on the size and nature of the practice (e.g., whether affiliated with a hospital or not). These data suggest that many clinics unaffiliated with hospitals may have more informal decision-making processes.

Thus, while Frith et al. [19] see ethical issues dichotomously as “settled” vs. “controversial”, with providers drawing boundaries between these two domains, the present data highlight other critical aspects of these issues. Certain ethical questions may appear “settled” in several clinics in the UK, but may remain more “controversial” elsewhere. Wide differences may also exist between clinics — especially in countries with less regulation. Ehrich et al. [15], for instance, argues that in the one UK clinic they observed, sex selection of embryos for nonmedical reasons remained very contentious, even though HFEA had banned it [30]. Hence, examination of *which* issues remain controversial and why, and how providers address them is critical.

Moreover, certain ethical issues, though seen as “settled” by providers, as Firth et al. suggest [19], can nonetheless pose a wide range of difficulties. Informed consent, for instance, may be seen as “mundane” — a mere formality — but in fact raise many challenges concerning definitions and applications of “voluntariness” in particular situations. Though the need for informed consent may be “settled”, obtaining it adequately with specific patients can still pose dilemmas — e.g., regarding how thorough it needs to be. Providers may thus face challenges regarding not only *what* to do ethically, but exactly *how* to do it. In unconventional IVF arrangements, family members’ complete voluntariness is crucial, but can be hard to assess. Individuals may agree, but still seem reluctant. While ASRM addresses needs to prevent undue influence, pressure and coercion, the current data highlight difficulties entailed. Voluntariness is complex and multi-faceted, varying along a spectrum [31], and can be inherently hard to gauge [32], shaped by not only undue influence, but subtler cultural expectations. Pressures from friends or family, for instance, may be more perceived than real, but still mold decisions. Decision-making often involves persuasion and influences from others that need not invalidate voluntariness [31]. While coercion has been defined as a “threat” [33], other, deeply personal feelings and beliefs may exist about implicit as well as explicit obligations to family members, strongly affected by cultural values and expectations. Family members may agree to unusual procreative arrangements due not to threats or overt pressure, but to deeply-instilled internal senses of duty, and patients from other cultures may view these issues differently.

The current data thus highlight needs to probe not just the *process* of how ethically-sensitive issues are dealt with in general, but the specific *content* of these ethical issues — what specific types of controversial cases arise, of what they consist, why they are controversial, what specific ethical tensions are involved, and how providers perceive and address these, and may differ in doing so based on various personal and professional factors. Ever-widening ART demand, use and types, changing social and culture mores, and mounting costs of buying gametes will doubtlessly make such unconventional arrangements ever-more logistically possible and financially advantageous for patients, confronting providers with more types and numbers of such unanticipated clinical situations.

Ehrich et al. showed that providers face grey areas, but questions then arise regarding how clinicians in fact resolve these. The current data suggest that providers may do so by undergoing various *psychological* processes, shifting from an initial “gut reaction” to more careful considerations of the ethical principles involved. Providers do not always know in advance how to evaluate or weigh these issues. While Gerrits and Ehrich observed meetings, but did not interview providers individually, the current data, based on in-depth interviews, suggest how clinicians may personally feel uncertain, and undergoing complex personal, emotional and moral, not just cognitive processes. Providers may at first automatically feel that these prospective combinations violate mores and are unethical. By definition, these combinations, made possible by relatively new technologies, are not traditional, making them unfamiliar, and hence not the “norm” (i.e., what is commonly done), prompting clinicians’ initial moral discomfort (e.g., “yuck”). Yet certain arrangements may not be the norm, but still be ethical. In the past, for instance, many providers felt that “gut feelings” justified not treating gay, lesbian, transgender or single patients; but many clinicians came later to alter their attitudes. Mores and ethos (what is generally done) can differ from morals and ethics (what one should do). Many clinicians may thus at first feel that such arrangements do not “smell right”, and only subsequently, if ever, more fully and explicitly consider the issues, carefully analyzing the ethical principles involved.

In general, individuals can, psychologically, feel bodily disgust or discomfort that they rapidly apply to social situations [34]. Incest taboos, for instance, have ongoing psychological potency, even when they do not in fact apply [35]. Consequently, providers may initially feel that a request involves combinations of gametes from consanguineous individuals when that is not in fact the case. In certain situations, feelings of revulsion can be evolutionarily adaptive (e.g., permitting rapid decisions about threatening situations). When faced with uncertainty, individuals often make fast, emotional and instinctive decisions, rather than slow, logical and conscious ones [36]. But some providers might simply follow their emotional impulses without reflection, withholding treatments from patients who should, arguably, receive care. Over time, mores, and hence feelings of discomfort will surely continue to evolve among providers, patients, and much of society-at-large, underscoring the importance of clinicians being able to think through each of these types of combinations.

While Frith et al. [19] found that providers, when asked if they could name any “ethical issues” could not do so, the current data suggest that clinicians may feel personally troubled and “uncomfortable” by issues that they do not explicitly label as “ethical,” but that indeed embody underlying ethical tensions, generating this discomfort – e.g., balancing patients’ autonomy vs. possible future risks to the unborn child. Future research on providers’ views and decisions regarding ethical issues should thus ask providers about not just “ethical issues” they face, but clinical situations that make them feel “uncomfortable”. Future research should also interview, not just observe clinicians wrestling with ethical issues to understand the ways providers themselves perceive and make these decisions, and the prevalence, validity, reliability and mutability of “gut” feelings in ART as well as other medical decisions.

The current data also contribute to the past literature by suggesting several specific types of clinical situations that can pose dilemmas. While the prior literature has mentioned a few types of unconventional parental arrangements, primarily involving intrafamilial genetically-related third parties [4–6], the present data suggest that difficulties can arise concerning unusual combinations of *social* relationships as well (e.g., friends or ex-partners), and that clinicians may feel concerns about not only creating children, but *raising* them as well. Patients may also present difficult scenarios not only initially, but overtime, altering their plans after starting treatment, and posing such dilemmas only later. While patients described by Gerrits et al. were all established couples [18], those in the present study often were not, and instead ﻿had complex relationships that themselves raised ethical questions, underscoring needs to examine not only *which* specific procedures are requested (e.g., gender testing or sex selection), but *who* is requesting them.

Regarding ethical principles, while Ehrich et al. argued that “relational autonomy” [15] may be a more suitable model than individual autonomy, the current data highlight how providers in fact consider not just the autonomy of a single patient, but the autonomy, rights and well-being of others, as well as other principles, including potential risks and benefits. Though many physicians in various fields prioritize the autonomy of the individual patient, unconventional combinations of parents may involve four or more people — not only one patient, but frequently a spouse, and third party (e.g., family member or friend donating gametes, carrying the fetus, and/or raising the child) — and the future child. Indeed, a full bioethical framework, as described by Beauchamp and Childress, includes four essential principles, all of which need to be considered, depending on the specific case: not only autonomy (i.e., the rights of all parties involved), but beneficence and nonmaleficence (e.g., the benefits and risks to each of the individuals involved), and social justice (e.g., not unfairly discriminating against certain groups such as gay men, lesbians single parents by choice) [37]. Providers should thus try to think through each case carefully, analyzing whether and how each of these four basic principles apply, to arrive at logical, rigorous and consistent ethical responses. Yet the autonomy, rights and best interests of the individuals involved can be hard to assess and/or can conflict. Frith et al. [19] and Gerrits et al. [18] point out, for instance, that providers are concerned about the welfare of the child, but the present data highlight how clinicians may view, assess and weigh the child’s welfare against these other concerns in a variety of ways that differ, based on the particular type of case. Questions arise, too, of the professional responsibilities of providers, since infertility treatments may not involve a “disease” per se, but are, arguably, more “elective”.

These data have several implications for clinical practice, highlighting the importance of clinicians being as sensitive as possible to these issues, ensuring that potential donors understand both that they indeed have a choice, and that providers could decline on potential donors’ behalf to potential gamete recipients, offering alternative reasons. These findings underscore, too, the benefits of formal ethics committees. Providers who refuse to treat cases that colleagues may feel are ethical, should consider referring these patients to such colleagues. However, how often such referrals occur is unknown. If declining to provide treatment, clinicians must decide, too, what exactly to tell the patient — how to explain and justify these decisions. These data also suggest that providers may benefit from ethics training on how to assess these dilemmas systematically, applying and weighing relevant ethical principles, as outlined earlier.

The data also have key implications for future guidelines. Current guidelines in the US and in many other countries do not address several critical questions that arise clinically, leaving many ambiguities, and are thus of limited assistance to providers. For instance, while ASRM recommends “separate interviews and counseling of the involved parties,” [3] the present data underscore how these interactions can still pose challenges. Indeed, studies of living kidney donors show that only 42% of transplant programs disclosed all elements of informed consent to prospective organ donors, and only 30% discussed long-term risks [38]; and of these donors, 40% felt pressure to donate — that their families “expected” them to do so — though being given both opportunities to alter their decision, and confidentiality concerning their reasons for declining [39]. Nevertheless, despite such familial expectations to donate, potential donors can nonetheless decide voluntarily that they indeed wish to do so [31]. Donation of organs differs from that of eggs or sperm; organ donation is generally more invasive, but illuminates the complexities that can be involved. ASRM could potentially address exactly how much proof of informed consent and voluntariness is needed. Guidelines for organ transplantation, for instance, recommend a 2-week “cooling off” period before surgery, to give donors time to fully weigh all the issues, though only 11% of programs require this period [40]. ASRM could similarly add to its guidelines that, especially when questions arise about informed consent, physicians should allow donors several days to carefully consider these decisions.

While ASRM states that “Child-to-parent arrangements are generally unacceptable, and parent-to-child arrangements are acceptable in limited situations” [3], questions emerge — e.g., whether ﻿the guidelines should change to stipulate that such arrangements should in fact “always,” rather than “generally” be prohibited; and if not, what exceptions are permissible, and how these should be determined, especially given concerns about informed consent. ASRM states that doctors may decline to provide treatment “on the basis of well-substantiated judgments that those patients will be unable to provide minimally adequate or safe care for offspring”; and that practices “should develop written policies and procedures for making determinations to withhold services on the basis of concerns about the child-rearing capacities of prospective patients” [41]. But how these judgements are or should be made is unclear. ASRM states that providers can reject gestational carriers due to “current marital or relationship instability” [42], but dilemmas arise of exactly how stable relationships, for parents as well, need to be.

Guidelines can address, too, arragements of friends and other social relationships, appropriate roles of physicians’ financial motives in these decisions, and how much providers should consider child-raising. Though ASRM has recommended that clinics develop written policies and procedures, questions remain regarding whether and how exactly providers do, and should proceed.

These findings highlight needs, too, for future research to investigate these views and experiences further among larger samples — e.g., how often clinicians confront each of these various types of arrangements, and agree or refuse treatment; how they decide and why; how often and in what ways clinics in fact develop written policies and procedures; what these policies say; how often providers follow these; when and how often providers balance emotional disgust vs. ethical principles; what clinicians say to patients when refusing to offer treatment; how often providers then refer these patients elsewhere; and what factors (e.g., provider age, gender, training, religion, and geographic location) affect clinicians’ decisions. ASRM should encourage, and the Society for Assisted Reproductive Technology can facilitate or encourage collection of data addressing at least some of these vital questions.

These data have implications, too, for understanding how clinicians make ethical decisions in medicine more broadly — e.g., when else providers draw on the “yuck” response, whether and how often they simply follow, or instead evaluate this response, how they assess it (e.g., using what criteria), and with what results.

The present data shed important light as well on recent questions concerning the interrelationships between research in bioethics ﻿and﻿ in moral and cognitive psychology [14, 19]. The current data suggest that critical questions emerge concerning when exactly individuals, in this case physicians, change from “fast” to “slow” thinking about complex moral issues — how long this change takes, and what factors, if any, may facilitate or impede it. These data thus suggest a research agenda these data — to examine whether individuals are able to overcome feelings of disgust regarding untraditional combinations of parents or other ethically controversial areas of medicine, and if so, how and when; and whether competing financial or other factors counter such “yuck” responses, and if so, how much. Further studies can probe, too, whether, over time, feelings of disgust lessen, disappear, co-exist, are wholly reversed (such that individuals instead come to follow "slow" thinking), or are merely countered by other considerations (e.g., “I still disapprove of certain behaviors, but will benefit financially, and will thus accept them”). Investigators can explore how feelings of disgust are related to final treatment decisions — whether the outcomes of “fast” thinking (e.g.., disgust) that individuals overcome differ from the outcomes of “slow” thinking, and whether aspects of the controversial treatment itself matter. Researchers can examine, for instance, how providers and the general public view potential unconventional parenting combinations— how many and which respondents would offer treatment, and how “yuck” responses vary in strength.

These data have several possible limitations. The sample size is adequate for qualitative studies designed to identify key themes and issues that appear, but not for statistically analyzing how various groups differ (e.g., male vs. female physicians, or mental health vs. other providers); however, further research can probe using domains with more participants. Still, clinicians, in particular, are very busy and increasingly difficult to recruit for research [43, 44]. Difficulty recruiting larger numbers of provides doubtlessly contributes to the lack of previous research on these key areas, and to the value of the current data. These data also arguably have as well a certain face validity in elucidating the challenges that many clinicians confront,.

## Conclusions

These interviews shed vital light on how clinicians confront critical dilemmas and make decisions about whether to treat unusual familial combinations of patients using ART; and have important implications for enhancing practice, policies, education and research.

## Abbreviations

ART: Assisted reproductive technology
ASRM: American Society for Reproductive Medicine
ESHRE: European Society of Human Reproduction and Embryology
HFEA: Human Fertilisation and Embryology Authority
IVF: In vitro fertilization
MD-PT: Physician-patient
MDs: Physicians
NHS: National Health Service
OP-PTs: Other provider-patients
OPs: Other providers
PTs: Patients
